# The Diagnosis of Meningitis

**Published:** 1899-12-09

**Authors:** 


### THE DIAGNOSIS OF MENINGITIS.

There are few things more difficult than to make a sure and satisfactory diagnosis in certain cases of meningitis. Addressing the Birkenhead Medical Society on this subject, Dr. Barr said that it might be supposed that a disease with such pronounced symptoms as meningitis would be always easily recognised, but such is not the case, for many of the most prominent cerebral symptoms are common to this and to other acute diseases. Headache and vomiting for example, though spoken of as the classical symptoms, are not more marked in meningitis than in typhoid and several other specific fevers. The headache has been made so much of by text-book writers that the medical man almost expects to see it, and because the patient is not shouting "Oh! my head," and writhing in agony, he doubts whether he has to do with the headache of meningitis. But the patient is really too ill to complain, he knows that the noise of his own voice as well as that of his questioner aggravates his sufferings. Again, there may not be much vomiting, for the appetite may be poor, and there may not be much to vomit. Among other early signs may be mentioned moderate pyrexia, delirium, stupor, occasionally general convulsions, irregularity of pupils, retraction of the head, retention of urine, &c. One of the earliest and most persistent symptom in some cases, especially in meningitis of the convexity, is general hyperalgesia, so that when a limb is firmly grasped the patient winces. In the same way there is apt to be intolerance of light and sound. Then one must consider the presence of the known causes or associates of the disease in its different forms. Purulent meningitis is associated with middle ear disease, necrosis of bone, abscess, septicæmia, and frequently with cerebro-spinal meningitis. Simple or plastic meningitis may be connected with pneumonia, rheumatism, or some of the specific diseases. Tuberculous meningitis is most common in children under ten years of age, but may occur in adults, especially in connection with tuberculous disease of the lungs, but after 40 years of age it is very rare. A common difficulty is the diagnosis between meningitis and typhoid fever, but in the early stages this ought not to be a great one. In typhoid the patient is not specially sensitive to external impressions; there is no intolerance of light or sound, no hyperalgesia, and he makes no attempt to remove the sordes from his teeth, or the dried secretion from the nose. In meningitis, on the other hand, and especially in meningitis of the convexity, marked general hyperalgesia and augmentation in all the reflexes are among the earliest symptoms. The patient is irritable and restless, picks at his nose and lips, throws his arms about in an aimless manner, perhaps gets out of bed without knowing why. So great is the hyperalgesia that, although the little patient may be lying apparently asleep, with eyes half open, the slightest movement or handling makes him scream out. The knee, elbow, and wrist jerks are generally well pronounced, and there may be well marked ankle and knee clonus. In typhoid fever, on the other hand, the deep reflexes are only exaggerated in severe cases and at a later stage of the disease. Apical pneumonia might be mistaken for meningitis, but physical examination and observation of the pulse respiration ratio will clear the matter up. Influenza of cerebral type may for a time simulate meningitis, but the symptoms quickly pass off, and the same may be said in regard to other acute specific diseases. Then comes the question of lumbar puncture, in regard to which it may be said that if a positive result is obtained—that is, if the micro-organisms are found which are associated with meningitis—the diagnosis is established; but unfortunately in many cases, even of purulent meningitis, the fluid is clear and no organisms are discovered. Widal's reaction may prove of service, but not in the early stage of typhoid fever.

